# Microwave‐Assisted Preparation of Hydrogel‐Forming Microneedle Arrays for Transdermal Drug Delivery Applications

**DOI:** 10.1002/mame.201500016

**Published:** 2015-03-23

**Authors:** Eneko Larrañeta, Rebecca E. M. Lutton, Aaron J. Brady, Eva M. Vicente‐Pérez, A. David Woolfson, Raghu Raj Singh Thakur, Ryan F. Donnelly

**Affiliations:** ^1^ Chair in Pharmaceutical TechnologySchool of PharmacyQueens University Belfast, Medical Biology Centre97 Lisburn RoadBelfastBT9 7BLUK

**Keywords:** crosslinking, hydrogels, microneedle, microwaves, transdermal drug delivery

## Abstract

A microwave (MW)‐assisted crosslinking process to prepare hydrogel‐forming microneedle (MN) arrays was evaluated. Conventionally, such MN arrays are prepared using processes that includes a thermal crosslinking step. Polymeric MN arrays were prepared using poly(methyl vinyl ether‐alt‐maleic acid) crosslinked by reaction with poly(ethylene glycol) over 24 h at 80 °C. Polymeric MN arrays were prepared to compare conventional process with the novel MW‐assisted crosslinking method. Infrared spectroscopy was used to evaluate the crosslinking degree, evaluating the area of the carbonyl peaks (2000–1500 cm^−1^). It was shown that, by using the MW‐assisted process, MN with a similar crosslinking degree to those prepared conventionally can be obtained in only 45 min. The effects of the crosslinking process on the properties of these materials were also evaluated. For this purpose swelling kinetics, mechanical characterisation, and insertion studies were performed. The results suggest that MN arrays prepared using the MW assisted process had equivalent properties to those prepared conventionally but can be produced 30 times faster. Finally, an *in vitro* caffeine permeation across excised porcine skin was performed using conventional and MW‐prepared MN arrays. The release profiles obtained can be considered equivalent, delivering in both cases 3000–3500 μg of caffeine after 24 h.
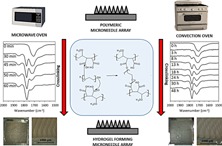

## 1 Introduction

Microneedle (MN) arrays are minimally‐invasive devices composed of micron‐size needles arranged in rows on a solid support. These devices are able to painlessly by‐pass outer skin barrier layer, the *stratum corneum*, the principal barrier to transdermal drug penetration. Consequently, these devices can be used to deliver drugs to the deeper layers of the skin, from where they can be absorbed directly into the systemic circulation.^1^ Currently, these systems are attracting substantial interest due to their ability to transform the applicability of transdermal drug delivery.^2^, ^3^, ^4^, ^5^

Microneedle arrays can be made from different materials, primarily silicon, metal, and polymers.^4^ Among polymeric MN, the hydrogel forming MN array system is of particular interest.^5^, ^6^, ^7^, ^8^, ^9^

Hydrogels are hydrophilic polymers interconnected by chemical and/or physical crosslinking, forming a three‐dimensional structure that is able to absorb large amounts of water.^10^, ^11^ These materials can be prepared using different crosslinking methods,^12^ with the main method using a thermal treatment step^6^, ^13^ with relatively high temperatures for prolonged periods of time.

An interesting alternative to the use of the standard thermal treatment is the use of microwave radiation. A wide variety of polymers have been synthesized using microwave radiation^14^, ^15^ including hydrogel preparations.^16^ Microwave assisted processes offer a series of advantages in contrast to conventional thermal processes. It combines a low energy consumption (green process) with a facile synthetic procedure that allows limited side reactions.^16^, ^17^, ^18^ In addition, microwave synthesis has higher reaction rates and yields in shorter periods of time.^14^, ^19^ All these features makes microwave‐assisted hydrogel synthesis a strong candidate for both laboratory and industrial applications.

To date, one of the main issues in MN technology is the development of a reliable scale‐up process.^20^, ^21^, ^22^ A microwave assisted process can be readily introduced into the development of an industrial manufacturing process for hydrogel‐forming MN. Thus, in this study the use of microwave radiation in the crosslinking process of hydrogel forming MN array is reported. These MN arrays were characterized and compared with those crosslinked using a conventional heating process.

## 2 Experimental Section

### 2.1 Materials

Gantrez^®^ AN‐139 (methylvinylether and maleic anhydride copolymer) (M_w_ = 1.0 × 10^6^ Da) and Gantrez^®^ S‐97 (acid form of methylvinylether and maleic anhydride copolymer) (M_w_ = 1.2 × 10^6^ Da), were provided by Ashland (Tadworth, Surrey, UK). Poly(ethyleneglycol) (PEG) 10,000 Daltons was obtained from Sigma‐Aldrich (Poole, Dorset, UK). Parafilm^®^ M, an olefin‐type flexible thermoplastic sheet (127 μm thickness), was obtained from Brand GMBH (Wertheim, Germany).

### 2.2 Methods

#### 2.2.1 Preparation of MN Arrays Using the Conventional Oven Assisted Process

Aqueous blends containing Gantrez^®^ S‐97 (20% w/w) and PEG 10,000 (7.5% w/w) were used to fabricate MN. This formulation was poured into laser‐engineered silicone micromould templates, centrifuged for 15 min at 3,500 rpm and allowed to dry under ambient conditions for 48 h.^23^ Finally they were placed inside a convection oven at 80 °C. In order to study the influence of the crosslinking degree in the performance of MN arrays, different curing times were used. All the arrays contained 19 × 19 needles. The dimensions were: 600 μm needle height, 300 μm width at the base and 50 μm interspacing. Some experiments were performed using baseplates (BP) instead of MN arrays. BP were prepared following the MN preparation process, but using moulds with no templates inside.

#### 2.2.2 Preparation of MN Arrays Using the Microwave‐Assisted Process

The MN and BP prepared following the microwave (MW) assisted process were manufactured following the same basic process previously described but with some variations. Instead of placing the moulds in a convection oven for the crosslinking process, the MN arrays were removed from the moulds, their side walls were removed and, finally, they were placed in the middle of the oven cavity in a Panasonic NN‐CF778S MW oven without a turntable (Panasonic UK Ltd, Bracknell, UK). The MN were crosslinked using different reaction times with the oven at the highest output power (1000 W).

#### 2.2.3 Infrared Measurements

Attenuated total reflectance (ATR)‐Fourier transform infrared (FTIR) spectroscopy was used to evaluate the crosslinking degree of Gantrez^®^/PEG polymer films and MN arrays. The IR spectra were recorded at room temperature using a FTIR Accutrac FT/IR‐4100 Series (Jasco, Essex, UK) equipped with MIRacle™ software between 4000–400 cm^−1^ with a resolution of 4.0 cm^−1^. The obtained spectra were the result of averaging 64 scans.

The crosslinking degree of the arrays was evaluated using the area under the different carbonyl peaks, the carbonyl peak of the Gantrez^®^ acid groups (A_A_) ca. 1720 cm^−1^, the carbonyl peak of ester groups formed between Gantrez^®^ and PEG (A_E_) ca. 1770 cm^−1^ and the carbonyl peak of the anhydride peaks formed between adjacent Gantrez^®^ acid groups (A_AN_) ca. 1850 cm^−1^. In order to follow the crosslinking reaction a factor called Crosslinking Factor (CF) (Equation 1) was calculated. This factor is proportional to the crosslinking degree.

(1) $$\text{CF}=A_{E}/(A_{A}+A_{E}+A_{\text{AN}})$$

#### 2.2.4 Baseplate Fracture Force

The break strength of MN base plates were evaluated using a TA.XT‐Plus Texture Analyser (Stable Micro Systems, Surrey, UK) in compression mode. Base plates were prepared in the same way as MN arrays, but using moulds with no needle cavities. MN base plates were placed on two aluminium blocks and tapered aluminium probe (5.5 cm in length with a blunt end of radius 1.0 mm) was moved towards the MN base‐plates. The probe moved at a speed of 2 mm s^−1^ with a maximum distance of travel of 5 mm. The baseplate failure force was assumed to be the peak maximum of the force‐distance curve.

#### 2.2.5 Axial Compression Force

A known load was applied to the MN arrays in axial compression (i.e., force applied perpendicular to the needle base) using a TA.XT‐plus Texture Analyser (Stable Micro Systems, Surrey, UK). MN arrays prepared with different crosslinking degrees were attached to the moving test probe of the equipment using double‐sided adhesive tape. The MN arrays were then pressed against a flat block of aluminium at a rate 0.01 mm/s until a maximum force of 290 N per array was applied. Pre‐test and post‐test speeds were 1 mm/s and 10 mm/s respectively and the trigger force was set at 0.001 N. The compressive stiffness was determined using Hooke's Law, which states that deformation is directly proportional to force. As such, the stiffness was calculated from the gradient of the linear portion of the force‐distance plot; this was done using Equation 2 and the mean calculated.

(2) $$\text{Compressive}\;\text{Stiffness}=(F_{2}-F_{1})/(S_{2}-S_{1})$$

where F_1_ and S_1_ are initial force and initial displacement values respectively in the linear plot while F2 and S2 are the final force and final displacement values.

#### 2.2.6 Insertion of MN Arrays

Parafilm^®^ M (PF) film was used as a skin simulant for MN insertion studies as described previously.^24^ A sheet of Parafilm^®^ was folded to get an 8‐layer film (≈ 1 mm thickness) and placed on a sheet of expanded poly(ethylene) for support. MN arrays were inserted using the Texture Analyser, with the probe lowered onto the artificial membrane at a speed of 0.5 mm s^−1^ with an exerted force of 40 N per array held for 30 s. Once the target force was reached, the probe was moved upwards at a speed of 0.5 mm s^−1^. The MN arrays were removed from the polymeric sheet after insertion, the PF sheet unfolded and the number of holes in each layer was evaluated using a Leica EZ4 D digital microscope (Leica, Wetzlar, Germany). In order to ease the detection of the created holes in the PF layers, the sample was placed between two polarizer filters. The thickness of each PF layer was determined previously (126 ± 7 μm)^24^ and was used to calculate the percentage of MN inserted as a function of the depth.

#### 2.2.7 Swelling Kinetics

Baseplates (1.0 × 1.0 cm) were weighed as m_o_ and then swollen in pH 7 phosphate buffer solution (PBS) for 24 h at room temperature. At regular intervals, the films were removed, dried with filter paper to eliminate excess surface water and weighed as m_t_ (hydrogels). The percentage swelling, was calculated, respectively, by using Equation 3.^25^

(3) $$\%\;\text{Swelling}=(m_{t}-m_{o})/m_{o}$$

#### 2.2.8 *In Vitro* Drug Permeation Studies Using Franz Cells

The diffusion of caffeine through microwave (45 min at 1000 W) and oven (24 h at 80 °C) crosslinked MN arrays across dermatomed neonatal porcine skin^26^ was investigated *in vitro* using modified Franz diffusion cells, as described previously.^6^ Skin samples were obtained from stillborn piglets and excised immediately (<24 h after birth). Using an electric dermatome (Integra Life Sciences™, Padgett Instruments, NJ, USA) the skin was trimmed to a 300–350 μμm thickness,frozen and stored at −20 °C until further use. Shaved skin samples were pre‐equilibrated in phosphate buffered saline (PBS) pH 7.4 (receptor medium) for at least 15 min before use. A circular piece of the skin was placed and secured to the donor compartment of the diffusion cell using cynoacrylate adhesive, with the *stratum corneum* side facing the donor compartment. MNs were inserted using manual force with a syringe plunger. After insertion the patches containing caffeine were attached to the upper baseplates of hydrogel‐forming MN. Caffeine patches were prepared using a casting method from aqueous blends of 10% w/w Gantrez^®^ AN‐139, 5% w/w tripropyleneglycol methyl ether and 3% of caffeine. Once MN arrays were in place, donor compartments were mounted onto the receptor compartments of Franz cells. The patch and the MN array were kept in place during the experiment by application of a metallic weight to their upper surface. Using a long needle, samples (0.30 ml) were extracted from the receptor compartment at defined time intervals and replaced with an equal volume of receptor medium. The concentration of caffeine in the receiver compartment were determined using HPLC.

Similarity factor, *f*_2_, is one of the statistical tools to compare dissolution profiles of solid oral dosage forms.^27^ Additionally, this factor has been used in many studies to compare permeation profile of different transdermal patch formulations.^28^, ^29^, ^30^ Two curves can be considered comparable when *f*_2_ value is larger than 50.^31^ The similarity factor (Equation. 4) is a logarithmic transformation of the sum‐squared error of differences between the fraction of release between the test, *T_t_* and reference *R_t_* over all time points, *n*.

(4) $$f_{2}=50\cdot \text{log}\left\{{\left[1+\left(\frac{1}{n}\right)\sum_{j=1}^{n}{{(R}_{t}-T_{t})}^{2}\right]}^{-\text{0,5}}\cdot 100\right\}$$

#### 2.2.9 Caffeine Quantification Method

Caffeine analysis was performed using RP‐HPLC (Agilent 1200^®^ Binary Pump, Agilent 1200®, Standard Autosampler, Agilent 1200^®^ Variable Wavelength Detector, Agilent Technologies UK Ltd, Stockport, UK) with UV detection at 273 nm. The column was a C18 (4.6 mm × 100 mm, 3.5 μm packing) XSelect CSH C18^®^ (Waters Associates, UK) analytical column fitted with a guard column of matching chemistry. The mobile phase consisted of an isocratic ratio of 74% v/v of a 10 mM Ammonium Acetate Buffer and 26% v/v methanol. The injection volume and flow rate were 20 μl and 1 ml/min, respectively. The column was kept at a constant temperature of 25 °C. The chromatographs obtained were analysed using Agilent ChemStation^®^ Software B.02.01.

#### 2.2.10 Statistics

All data were expressed as mean ± standard deviation. Data were compared using a paired, two‐tailed Student's *t*‐test when comparing two means and One‐Way Analysis of Variance (ANOVA), with Tukey's HSD *post‐hoc* test for more than two means. In all cases, *p* < 0.05 was the minimum value considered acceptable for rejection of the null hypothesis.

## 3 Results and Discussion

### 3.1 Crosslinking of MN Arrays Using Conventional Oven Assisted Process

**Figure 1:**
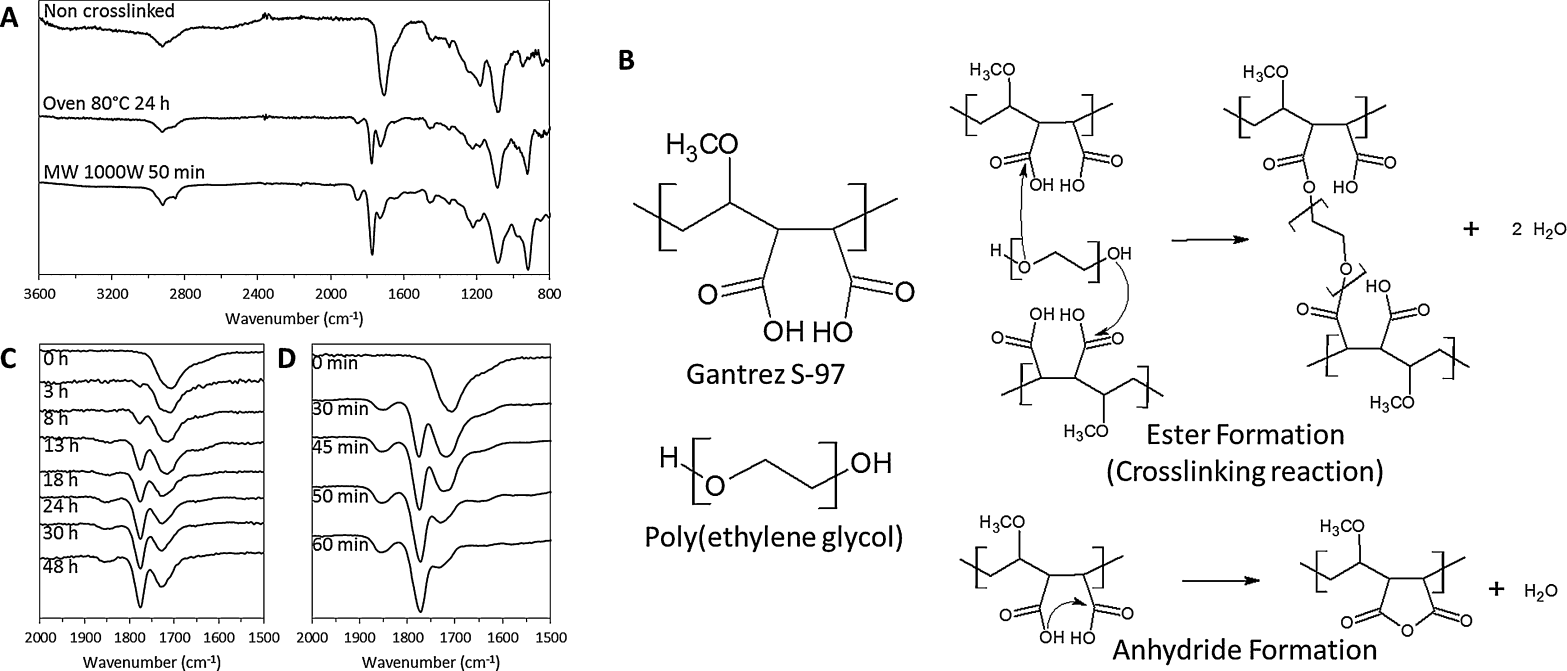
FTIR‐ATR spectra of non crosslinked, conventionally crosslinked and MW crosslinked hydrogels (a). Chemical structures and proposed chemical reactions that take place during the crosslinking process (b). FTIR‐ATR carbonyl region for different crosslinking times using the conventional (c) and the MW (d) crosslinking methods.

When preparing hydrogels, the degree of crosslinking is a critical issue that directly affects different properties of the material, especially the swelling kinetics.^25^, ^32^ This is a key aspect when hydrogels are prepared for drug delivery purposes.^33^, ^34^, ^35^ For this purpose infrared spectroscopy can be used to evaluate the crosslinking degree in Gantrez^®^ hydrogels. The main difference between the infrared spectra of oven crosslinked and non‐crosslinked hydrogels can be found in the carbonyl region (2000—1500 cm^−1^) Figure 1(**a**). The non‐crosslinked hydrogel presents only a single peak (ca. 1720 cm^−1^) that can be attributed to the acid carbonyl groups of Gantrez^®^ S‐97 Figure 1(b). On the other hand, the spectra of the hydrogel treated at 80 °C show three different carbonyl peaks. The first at *ca*. 1850 cm^−1^ can be attributed to the formation of anhydride groups between adjacent acid groups in the Gantrez^®^ S‐97 chains Figure 1(b). The second carbonyl peak (ca. 1770 cm^−1^) belongs to the ester carbonyl formed between the Gantrez S‐97 acid groups and the terminal hydroxyl groups form the PEG chains Figure 1(b). Finally, the third carbonyl peak (ca. 1720 cm^−1^), as pointed out above, corresponds to the acid carbonyl groups. As can be seen in Figure 1(c), the ester carbonyl peak intensity increases with crosslinking time using the conventional oven process at 80 °C.

**Figure 2:**
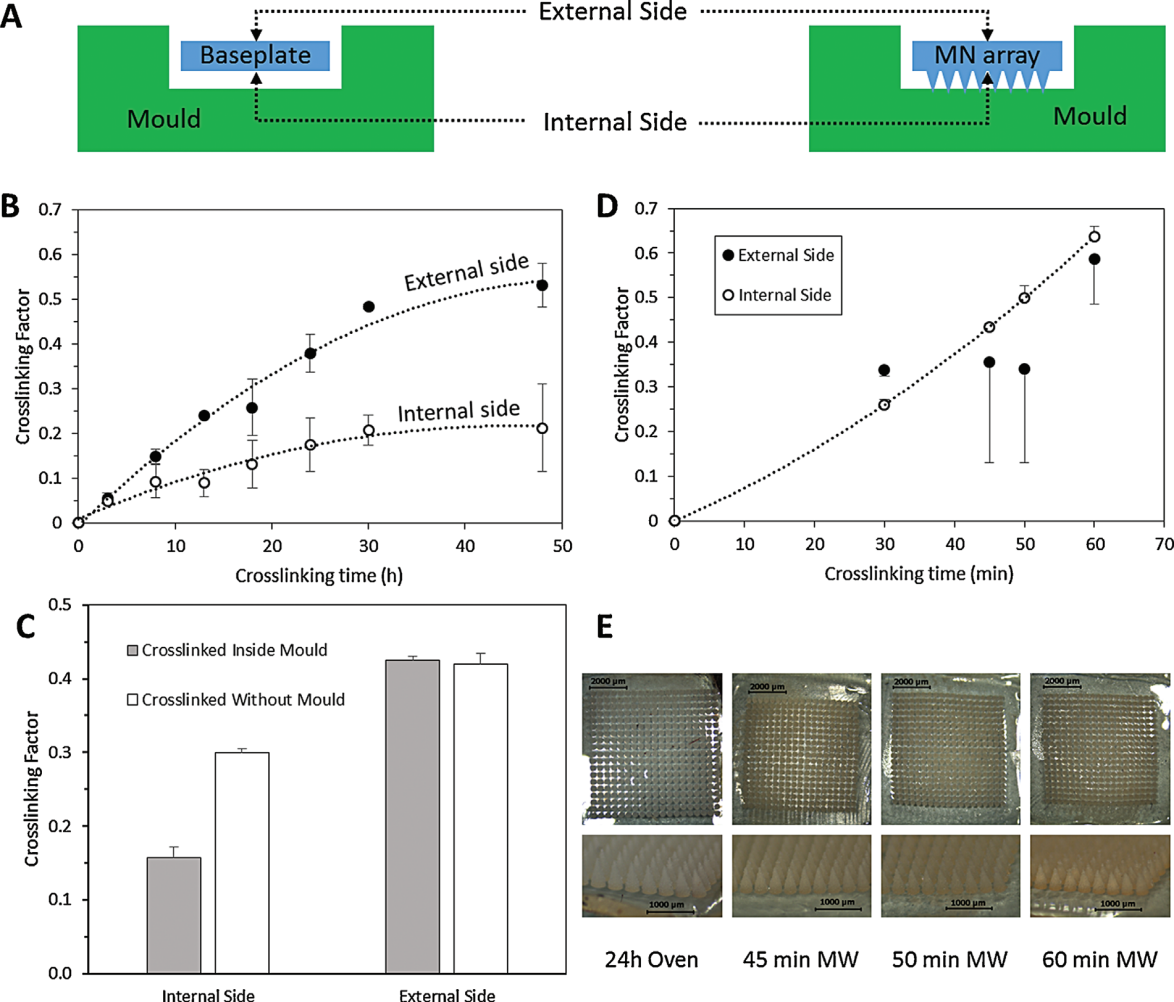
Diagram of baseplate/MN array inside a mould (a). Crosslinking factor as a function of the crosslinking time for hydrogels crosslinked using the conventional oven crosslinking method (b). Effect of the moulds in the crosslinking factor after conventional crosslinking method (c). Crosslinking factor as a function of the crosslinking time for hydrogels prepared using the MW crosslinking method (d). Comparison between MN arrays prepared using different crosslinking times using the MW process and a MN array crosslinked during 24 h using the conventional method (e). (Means ± SD, *n* = 3).

Evaluating the different areas under the carbonyl peaks, a factor defined as “Crosslinking Factor” (Equation. 1) can be calculated and gives information about the crosslinking process as CF is proportional to the crosslinking degree. This factor was used previously to evaluate the esterification degree in other types of macromolecules such as pectins.^36^ The CF depends on the side of the BP/MN array inside the mould after the crosslinking process (Figure 2(**a**). Figure 2(b) shows the obtained CF for baseplates as a function of the crosslinking time at 80 °C. The surface that was in contact with the mould during all the drying and crosslinking processes (internal side) has a lower degree of crosslinking (lower CF value) than the external side. In addition, the crosslinking degree in both surfaces increases with the oven time until a plateau is reached for the higher crosslinking time samples. This can be explained by the thermal insulating nature of the mould that hinders the heating of the inner side of the array.

When the crosslinking temperature was reduced to 60 °C, after 24 h of treatment, the crosslinking degree of both sides of the baseplate were both around 0.03 (*p* = 0.0566). These values were lower than the ones obtained after 3 h of crosslinking at 80 °C (*p* < 0.05).

For MN arrays the measured CF values as a function of oven heating time were consistent with those obtained for the baseplates Figure 2(c). In addition they showed the same behavior with lower crosslinking degrees on the needles side (internal side) (*p* < 0.05).

Another .critical issue in the manufacturing process of MN arrays is the use of moulds during the crosslinking process. When the crosslinking process in the oven was carried out without moulds it can be seen that higher CF values in the needle side were obtained Figure 2(c). On the other hand, the obtained CF values for the outside part of the arrays crosslinked inside the moulds and with no moulds did not present significant differences (*p* = 0.5208). The MN arrays crosslinked inside the moulds present lower CF values in the needle side than those that were crosslinked without moulds. The temperatures reached in the inner side of the array should be lower than the outside because the moulds are made of silicone elastomer, a thermal insulating material. However, the inner side CF values for the MN arrays crosslinked without moulds were not as high as the outer side ones. The presence of residual amounts of water in the inner side could explain the difference in the degree of crosslinking. The needle side of the array should present slower drying kinetics, so a small amount of water is expected to be present. This water will hinder the esterification reaction.^37^ Taking into account all the data obtained, and given that and that CF is straight forward to calculate, it can be easily incorporated as a quality control measure for future scaled‐up MN manufacturing processes.

**Figure 3:**
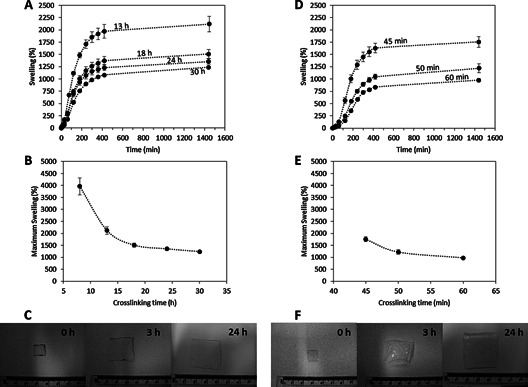
Swelling curves for hydrogels crosslinked using the conventional method for different crosslinking times (a). Maximum swelling obtained after 24 h as a function of the crosslinking time for hydrogels prepared using the conventional process (b). Pictures of hydrogels crosslinked during 24 h using the conventional method for different swelling times (c). Swelling curves for hydrogels crosslinked using the MW method for different crosslinking times (d). Maximum swelling obtained after 24 h as a function of the crosslinking time for hydrogels prepared using the MW process (e). Pictures of hydrogels crosslinked during 45 min using the MW method for different swelling times (f). (Means ± SD, *n* = 3).

The crosslinking degree affects the properties of these hydrogel materials, most importantly in respect of their swelling kinetics Figure 3(**a**). The BP prepared using lower crosslinking times had higher swelling percentages. Additionally, the crosslinking time can be easily related to the swelling kinetics and maximum swelling Figure 3(b). It is noticeable that the lower maximum water intake for the conventional hydrogels can be found for the hydrogels prepared using longer crosslinking times. Thus, the maximum swelling is lower for hydrogels with higher crosslinking densities. This is consistent with a previous study^25^ that evaluated the swelling kinetics of a similar type of hydrogel based on Gantrez AN‐139 rather than Gantrez S‐97.

**Figure 4:**
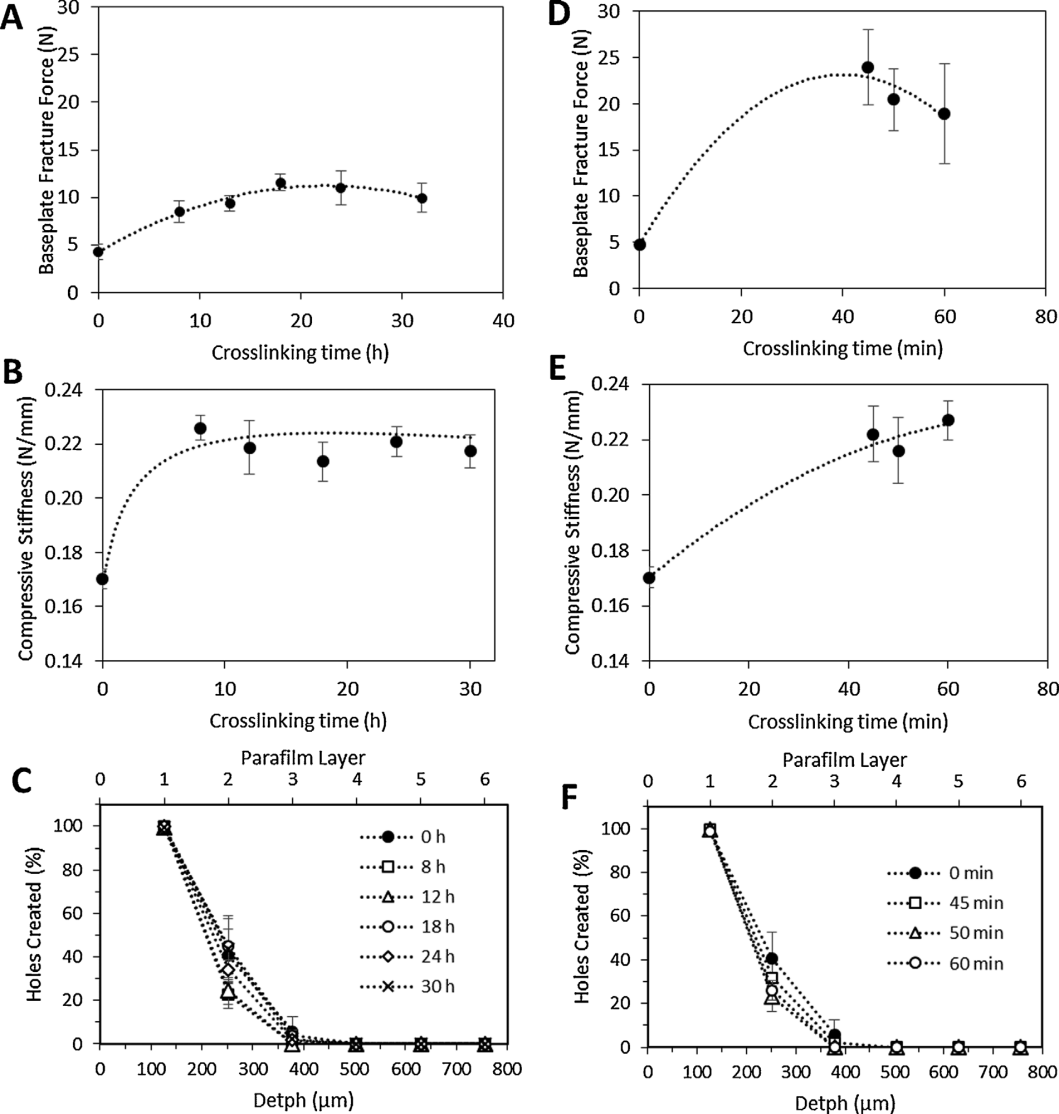
Baseplate fracture force (a) and MN compressive stiffness (b) as a function of the crosslinking time for hydrogels prepared using the conventional method (Means ± SD, n ≥ 3). Insertion profile in Parafilm for MN prepared using different crosslinking times following the conventional oven process (Means ± SD, n = 3) (c). Baseplate fracture force (d) and MN compressive stiffness (e) as a function of the crosslinking time for hydrogels prepared using the novel MW method (Means ± SD, *n* ≥ 3). Insertion profile in Parafilm® for MN prepared using different crosslinking times following the MW process (f). (Means ± SD, *n* = 3).

Subsequently mechanical properties of the material were evaluated. The mechanical resistance of the BP was tested by applying a three point bending test.^38^ The fracture force of BP increased slightly when the material was crosslinked Figure 4(**a**) (*p* < 0.05). Nevertheless there were no significant differences between the obtained fracture forces for the BP crosslinked during 18 and 30 h (*p* > 0.05).

After evaluating the crosslinking degree and the flexibility of the material the next step was to prepare MN arrays using this technique and evaluate their compression mechanical resistance and insertion properties. For the former, the compressive stiffness of the needles in the 19 × 19 array was evaluated as a function of the crosslinking time Figure 4(b). There were no significant differences between the obtained compressive stiffness for the crosslinked MN arrays (*p* = 0.3188). Nevertheless, when the MN arrays were not crosslinked the compressive stiffness had significantly lower values (*p* < 0.05). In conclusion, the mechanical properties of these materials were not influenced by the curing time Figures 4(a and 4b). Nevertheless once the material was crosslinked the mechanical properties of the obtained material were distinctly different to the mechanical properties of the non‐crosslinked MN arrays.

The insertion depth of the MN arrays was evaluated using the method proposed by Larraneta et al.^24^ using an artificial membrane Figure 4(c). MN mainly pierce the first two PF layers. Around 100% of the needles pierced the first PF layer while less than 50% pierced the second one. The obtained insertion profiles seem to be independent of the crosslinking time. This suggests that the insertion depth is more influenced by the needle density/design^39^, ^40^, ^41^ rather than by the crosslinking density of the material.

### 3.2 Crosslinking of MN Arrays Using Microwave Assisted Process

The issues involved in large‐scale production of MN products require careful consideration. Some of the required processes will add substantially to manufacturing costs.^42^ In the production of hydrogel forming MN arrays, there is normally a thermal process to crosslink the polymeric chains.^6^, ^13^ As an alternative to the conventional heating process, microwaves can generate heat directly within the sample and thus offer possible advantages of higher efficiency, faster production rate and lower capital costs.^15^, ^43^

The FTIR spectra for the MW crosslinked hydrogels showed the same typical carbonyl peaks that can be found in MN prepared using the conventional oven method Figure 1(a), 1(c) and 1d). However, these changes can be observed in around 60 min, rather than the 18—24 h required by conventional oven heating.

Figure 2(c) shows the obtained CF for baseplates as a function of the MW heating time. In general, a higher CF can be observed for higher crosslinking times. In this case, the higher CFs were obtained in the side of the BP that has been in contact with the mould. This CF values can be compared with the CF of the hydrogels prepared using the conventional process. As can be seen in Figure 2(a) and 2(c), using MW radiation to crosslink the hydrogels, similar crosslinking degrees using 45—60 min rather than 24 h can be obtained. The main difference is that the highest crosslinking degrees were obtained in the needle side of the array. This happens because the process is different than that of a conventional oven. As described above, the BP/arrays were removed from the moulds and then placed in the MW oven with the side that has been in contact with the mould facing up. This also explains the larger error bars for the CF in the lower side of the array as the MW distribution is not as homogeneous as it is in the side where the needles are placed. Nevertheless, as can be seen below, the swelling profiles Figure 3(d) and 3(e) presented reproducible results so the difference in the obtained CF can be due only to a very small region of the centre of the array. On the other hand, this behavior is not observed for the BP prepared using 30 min as crosslinking time. In this case, the crosslinking degree is lower in the inner side than in the outer one for the BP prepared and the error bars were smaller. This may be due to the lower temperatures reached in the array during the process.

The swelling profiles of BP prepared using the MW‐assisted process can be seen in Figure 3(d). As pointed out above, the BP prepared with longer crosslinking times presented higher percentages of swelling Figure 3(e). In addition, the swelling profiles were similar in all cases. Nevertheless, the form of the curves is slightly different than those of the BP prepared using the conventional oven process. They present a short latency period at the beginning. The samples prepared using 30 min as crosslinking time were not evaluated because some samples were very difficult to handle. Additionally, part of the arrays were dissolved throughout the swelling process when they were placed in the aqueous medium, indicating an incomplete crosslinking process.

Hydrogels prepared using MW with 45 min crosslinking duration presented the closest swelling profile to those crosslinked during 24 h following the conventional oven process previously described by Donnelly (Figures 3(c) and 3(f).^6^, ^7^, ^22^ Nevertheless the swelling profile for MW prepared hydrogels is slightly different to those prepared conventionally, showing short initial latency time (around the first 100 min) in the swelling profile.

The forces needed to break a BP as a function of the crosslinking time are shown in Figure 4(d). Again, there was a noticeable difference between the fracture forces for the non‐crosslinked and crosslinked BP(*p* < 0.05), although there were no significant differences in the fracture forces of the BP obtained using different crosslinking times (*p* > 0.05). Additionally, it is noticeable that the range of obtained fracture forces were higher than those obtained in the BP prepared with the conventional method (*p* < 0.05). This can be explained by taking into account that the force in the bending test was applied to the external side Figure 2(b) and 2(d).

Figure 4(e) shows the compressive stiffness of the needles as a function of the crosslinking time. As described for the oven‐prepared MN arrays, there were no significant differences between the obtained compressive stiffness for the MN arrays prepared with different crosslinking times (*p* = 0.4751). Nevertheless, there were significant differences between the non‐crosslinked and the rest of the evaluated MN arrays (*p* < 0.05). Additionally there were no significant differences for these values in comparison with those obtained for the BP prepared following the conventional crosslinking method (*p* = 0.4837).

Additionally, the insertion profiles of the MN prepared using the MW assisted method were evaluated Figure 4(f) using the Parafilm^®^ method.^24^ The insertion profiles seem to be equivalent to those of the conventional MN, with the needles piercing the first two PF layers (ca. 100% of the needles pierced the first PF layer and less than 50% pierced the second one). Thus, the crosslinking method did not affect the insertion profile of the MN arrays.

### 3.3 *In Vitro* Drug Permeation Studies Using Franz Cells

**Figure 5:**
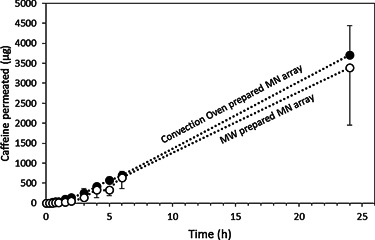
*In vitro* cumulative permeation results of Caffeine delivered from integrated hydrogel forming MN/caffeine patch across dermatomed neonatal porcine skin of 300 ± 50 µm thickness using conventional oven (24 h at 80 °C) (●) and microwave (45 min at 1000 W) () prepared MN arrays.

To determine the effect, if any, on drug release characteristics as a function of crosslinking method, a caffeine permeation study across neonatal dermatomed skin was performed. Figure 5 shows the *in vitro* permeation kinetics of caffeine from both types of MN arrays across dermatomed neonatal porcine skin. The caffeine permeation curves from both types of MN present the same profile. In addition, the final amount of caffeine permeated after 24 h can be considered equivalent. The amount of caffeine permeated after 24 h were, in both cases, around 35% of the amount present in the caffeine patch (36 ± 6% for conventional MN and 36 ± 12% for the MW‐prepared MN).

The *f*_2_ factor was calculated to compare all permeation profiles Equation (4). The obtained values were in all cases higher than 60, indicating that the caffeine permeation profiles through both types of MN can be considered comparable.^31^

This study shows that the permeation profile of caffeine from both types of formulation can be considered equivalent. Donnelly et al.^6^ previously demonstrated the ability of MN to deliver caffeine through the skin. The reported amount of caffeine permeated in that study was approximately 2.5 times lower than for the MW crosslinked array. This is consistent with caffeine loading being 3 times lower in that case.

## 4 Conclusion

In this work we have shown the advantages of a MW assisted crosslinking process in contrast with conventional thermal heating for the preparation of hydrogel forming microneedle arrays. The crosslinking time was reduced by a factor of approximately 30. Thus, the use of microwave radiation significantly reduces the time required for MN preparation and, in addition, microwave thermal processes are cheaper and quicker than conventional (oven) heating Therefore, the findings in this paper are of interest for MN crosslinking applications, leading to shorter, cheaper and greener manufacturing processes.
